# Smartphone-Based Pelvic Movement Asymmetry Measures for Clinical Decision Making in Equine Lameness Assessment

**DOI:** 10.3390/ani11061665

**Published:** 2021-06-03

**Authors:** Eva Marunova, Leea Dod, Stefan Witte, Thilo Pfau

**Affiliations:** 1 Department of Clinical Science and Services, The Royal Veterinary College, Hawkshead Lane, Hatfield AL9 7 TA, UK; ldod6@rvc.ac.uk (L.D.); tpfau@rvc.ac.uk (T.P.); 2 Tierklinik Schönbühl AG, Oberdorfstrasse 1, 3322 Schönbühl, Switzerland; stefan.witte@tierklinik-schoenbuehl.ch

**Keywords:** horse, lameness, smartphone, movement asymmetry, gait analysis

## Abstract

**Simple Summary:**

As visual evaluation of hindlimb lameness in the horse is challenging, objective measurements could aid clinical decision making. Our study investigated the association of pelvic movement asymmetry, recorded via a smartphone, with lameness scores of one experienced veterinarian. In general, cut-off points between lameness grades increased with increasing severity of lameness. Pelvic upward movement amplitude difference was the asymmetry parameter with the highest discriminative power based on subjective lameness scoring of a specialist veterinarian. This parameter classified a higher proportion of lame and non-lame horses correctly compared to the parameters assessing differences between pelvic vertical movement minima and maxima. Although the use of a smartphone measuring only the symmetry of pelvis cannot replace a full lameness examination, it presents a useful adjunct to subjective evaluation alone. Quantification of pelvic asymmetry with a smartphone may also be a useful tool in the context of tracking progress after a treatment or during the rehabilitation process.

**Abstract:**

Visual evaluation of hindlimb lameness in the horse is challenging. Objective measurements, simultaneous to visual assessment, are used increasingly to aid clinical decision making. The aim of this study was to investigate the association of pelvic movement asymmetry with lameness scores (UK scale 0–10) of one experienced veterinarian. Absolute values of pelvic asymmetry measures, quantifying differences between vertical minima (AbPDMin), maxima (AbPDMax) and upward movement amplitudes (AbPDUp), were recorded during straight-line trot with a smartphone attached to the sacrum (*n* = 301 horses). Overall, there was a significant difference between lameness grades for all three asymmetry measures (*p* < 0.001). Five pair-wise differences (out of 10) were significant for AbPDMin (*p* ≤ 0.02) and seven for AbPDMax (*p* ≤ 0.03) and AbPDUp (*p* ≤ 0.02). Receiver operating curves assessed sensitivity and specificity of asymmetry measures against lameness scores. AbPDUp had the highest discriminative power (area under curve (AUC) = 0.801–0.852) followed by AbPDMax (AUC = 0.728–0.813) and AbPDMin (AUC = 0.688–0.785). Cut-off points between non-lame (grade 0) and lame horses (grades 1–4) with a minimum sensitivity of 75% were identified as AbPDUp ≥ 7.5 mm (67.6% specificity), AbPDMax ≥ 4.5 mm (51.9% specificity) and AbPDMin ≥ 2.5 mm (33.3% specificity). In conclusion, pelvic upward movement amplitude difference (AbPDUp) was the asymmetry parameter with the highest discriminative power in this study.

## 1. Introduction

Assessment and grading of equine lameness can be a challenge for veterinarians since they rely on subjective visual recognition of changes in movement. As inter-observer agreement has been reported to be poor even for experienced equine veterinarians [1], objective kinematic measurements, in particular those recorded by inertial sensors, have been increasingly incorporated into lameness evaluations [2,3]. While these systems offer a high degree of accuracy and repeatability between trials [4], the equipment cost may be prohibitive for a wider use, for example to regularly monitor movement symmetry. A smartphone recording pelvic movement with its single built-in inertial sensor has been shown to deliver data with a precision comparable to that recorded by a multi-sensor specialist system [5]. Therefore, asymmetry measures obtained via a smartphone might be a useful adjunct to the lameness evaluation when more sophisticated equipment is not available. This might be particularly useful for assessing hindlimb movement asymmetries as hindlimb lameness tends to be more difficult to assess visually than forelimb lameness [6]. While tuber coxae movement (‘hip hike’) is often used to measure hindlimb lameness visually [7,8], motion analysis studies have established that quantification of hindlimb lameness can be achieved by measuring the vertical motion of the sacral bone alone [9,10]. The symmetry measures calculated from this anatomical location include the differences in maximum and minimum position of the pelvis between the two halves of the stride cycle (PDMax and PDMin) [11,12,13] and the difference between sacral upward movement amplitudes (PDUp) [9,10]. With regard to hindlimb lameness, guideline values for the cut-off for PDMax and PDMin between non-lame and lame horses have been established as 3 mm [4,12]. However, other studies of ‘owner-sound’ horses in regular work [14] and racehorses in training evaluated as sound by veterinarians [15] reported pelvic asymmetry values exceeding these guideline values, suggesting that horses with asymmetrical movement might not always have an underlying condition that could warrant a lameness investigation. It is also important to note that different technological solutions, for example, different inertial-sensor systems, may lead to differences between measurements due to different sensors and/or data processing [5,16].

While there is some evidence that objective asymmetry measures might correlate with subjective lameness scores [12,17], no reference ranges for different lameness grades have been proposed. Although knowledge of the discriminative power of the various asymmetry measures is limited [18,19], previous studies indicate that for hindlimb lameness, PDMin shows high sensitivity and specificity when compared with lameness scores of a group of veterinarians [18]. PDMin was also found to be a parameter showing the largest response per degree of visual lameness score after administration of diagnostic analgesia to a hindlimb [20]. To date, no cut-off points have been determined for PDUp with regard to distinguishing between lame and non-lame horses, and it might be useful to understand how this parameter compares to the more frequently utilised PDMin and PDMax.

The aims of this study were (a) to investigate sensitivity and specificity of three smartphone-derived pelvic movement asymmetry measures when compared to the subjective visual assessment of one expert veterinarian and (b) to quantify objective threshold values for different lameness grades based on this individual’s subjective assessment. We hypothesised that (a) PDMin would have higher discriminative power and (b) the asymmetry measures would have distinct thresholds that would increase with the severity of lameness.

## 2. Materials and Methods

This retrospective study was approved by the Social Science Research Ethical Review Board at the Royal Veterinary College (URN SR2019-0426).

### 2.1. Horses

Records of 301 horses assessed at a veterinary hospital (Tierklinik Schönbühl, Schönbühl, Switzerland) in Switzerland were included in this study. The mean age was 12.2 years with range 3 to 26 (age not recorded, NR, in 2 cases), and there were 165 geldings, 132 mares and 2 stallions (NR = 2). The majority of horses were warmbloods (*n* = 214) with a variety of other breeds also represented (21 cold bloods, 21 ponies, 18 Quarter Horses, 9 Arabians, 8 Thoroughbreds, 4 Cobs, 3 Andalusian, 1 Pinto, 1 Criolo and 1 NR). Horses were included in the study when two conditions were met: (1) hindlimb gait had subjectively been assigned a lameness grade and (2) pelvic gait symmetry had been measured. Not all horses included in the study were presented specifically for lameness investigation, hence the study population consisted of horses visually categorised as non-lame as well as lame.

### 2.2. Lameness Scoring

Lameness grades from 0 (non-lame) to 10 (non-weightbearing), UK scale [8], were based on visual observations of one board-certified veterinary surgeon (Dip. ACVS, Dip. ACVSMR). UK scale was chosen as it allowed us to relate the severity of lameness to an increase in asymmetry in trot on a straight line alone as opposed to observing the horse in different gaits and under different conditions.

### 2.3. Equipment and Setup

**Figure 1:**
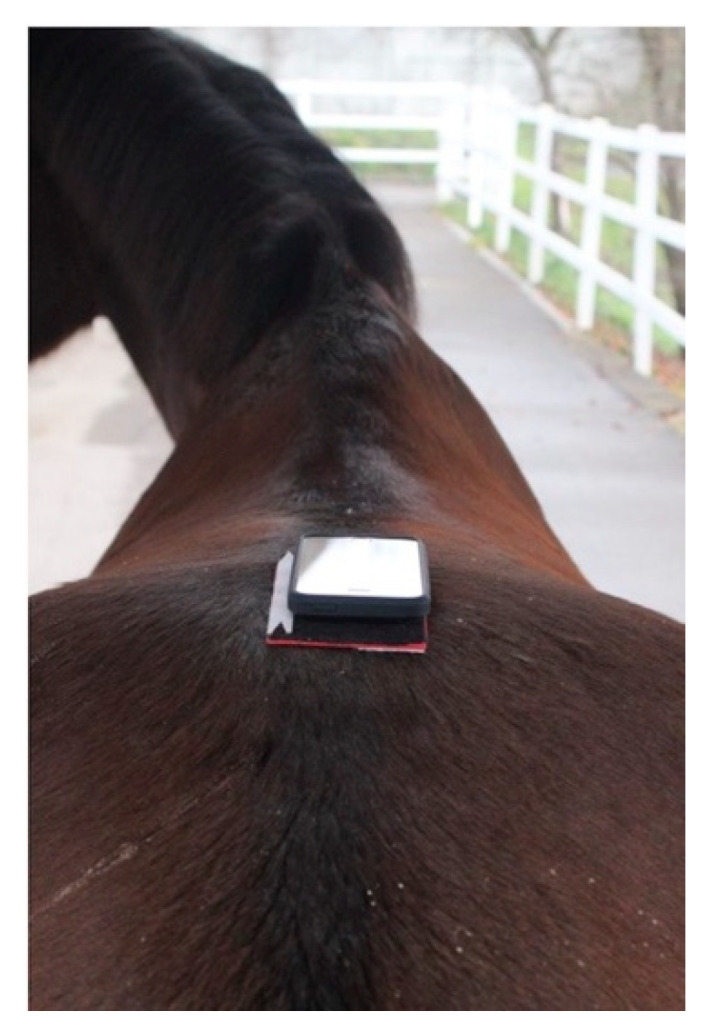
Image demonstrating the placement of the smartphone during data collection.

Each horse was equipped with an off-the-shelf smartphone (iPhone6, with internal inertial measurement unit: ± 16 times gravitational acceleration, ± 2000°/s; mass of device 129 g). The smartphone was attached onto a specially modified Velcro pad that was fixed centrally over the sacrum of the horse via double-sided tape (Figure 1). The Velcro pad had an 11-mm-wide strip on the left long side of the pad. This guided placement of the phone so that while the pad was attached centrally on the horse, the smartphone was placed 11 mm to the right of the centre [5], ensuring that the in-built accelerometer was positioned directly above the horse’s midline.

### 2.4. Data Collection

Data collection was performed using the iOS Application ‘SensorLog’ (sensorlog.berndthomas.net) available via the App Store. The software was configured to collect 3-axis acceleration (option ‘ACC’ providing x, y and z acceleration in multiples of gravitational acceleration) as well as orientation data (option ‘DM’ providing roll, pitch and yaw in radians) at a sample rate of 50 Hz per individual channel. Data collection was started manually before the device was attached to the horse and stopped manually immediately after the horse had performed the required exercise and the device had been removed from the horse.

All data were collected by an experienced board-certified equine specialist at a consistent venue (Tierklinik Schönbühl, Schönbühl, Switzerland) over a period of three years (2016–2019). Each horse was evaluated only once under the following protocol: (1) The horse was trotted by the same handler in hand in a straight line on a hard surface for approximately 25 m away from and then towards the assessing veterinarian. (2) The smartphone was then removed from the horse and the veterinarian assigned his subjective lameness grade before uploading the data from the smartphone to a Windows-based computer for asymmetry analysis.

### 2.5. Data Processing

Using custom-written software (MATLAB, The Mathworks, Natick, MA, USA), portions of the acceleration data representing steady state trot were identified visually by evaluating the timing and amplitudes of vertical acceleration of the phone. Therefore, data recorded before the horses started to trot and during the turn were excluded from the analysis. The identified steady state trot portions were then further automatically segmented into individual strides following published procedures [21]. Median values were calculated over all stride cycles for the following movement asymmetry values derived from vertical pelvic motion for each individual stride cycle: PDMin: differences between the vertical minima reached during left and right hind stance; PDMax: differences between the vertical maxima reached after left and right hind stance [11]; and PDUp: difference between the vertical movement amplitudes from the minimum at mid stance to the maximum in the aerial phase between the left and right half of the stride cycle [10,22]. The median values obtained for each horse were converted into absolute values (AbPDMin, AbPDMax, AbPDUp) in order to investigate the amount of movement asymmetry measured for each horse independent of the direction of asymmetry.

Statistical analysis was carried out using SPSS software (IBM Corp, Armonk, NY, USA, version 26) and RStudio (RStudio Inc., Boston, MA, USA, version 3.6.1). The level of significance was set at *p* < 0.05. Descriptive statistics and box plots were obtained, and Shapiro–Wilk normality tests were carried out for AbPDMin, AbPDMax and AbPDUp. As the data were not normally distributed (*p* < 0.001 for all three measures), Kruskal–Wallis tests with pairwise Bonferroni post-hoc comparison were implemented to analyse differences between lameness grades. Horses with lameness grade ≥ 5 were excluded from all but basic data analysis due to an insufficient number of observations (grade 5: *n* = 4; grade 6–10: *n* = 0).

Receiver operating characteristic (ROC) curves were obtained based on absolute values of the asymmetry measures and subjective lameness scores. True positive rate (sensitivity) indicates the proportion of horses that were correctly classified as positive, i.e., falling into a given lameness grade or higher, based on movement asymmetry measures. False positive rate (1-specificity) indicates the proportion of horses that were incorrectly classified as positive, i.e., classified into the given lameness grade or higher based on movement asymmetry, when they were non-lame or showed a lower lameness grade. Area under the curve (AUC) was calculated to determine the quality of each asymmetry measure in terms of their ability to differentiate between horses at or above a given lameness grade and horses below a given lameness grade. Cut-off points for different lameness grades were investigated based on minimum requirements for: (a) specificity and (b) sensitivity.

## 3. Results

The *n* = 301 horses were, based on visual assessment, classified into the following lameness grades: grade 0 (non-lame): *n* = 108 (36%); grade 1: *n* = 54 (18%); grade 2: *n* = 60 (20%); grade 3: *n* = 32 (11%); grade 4: *n* = 43 (14%); and grade 5: *n* = 4 (1%). No horses were classified as grade 6 or above. Most horses displayed asymmetries which caused concurrent non-zero differences in pelvic minima and maxima, i.e., few horses displayed purely ‘impact’ (PDMin) or ‘push off’ (PDMax) asymmetries (Supplementary data S1).

**Figure 2:**
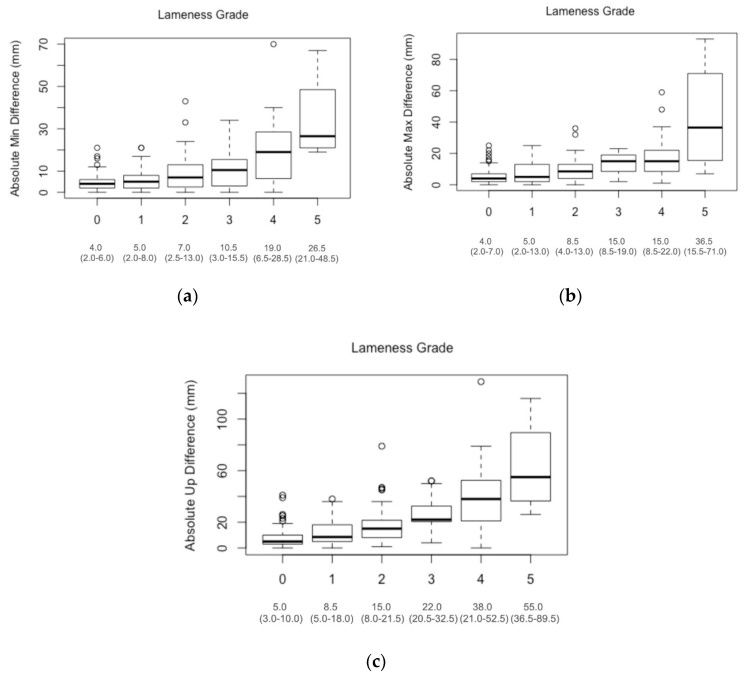
Box plots illustrating the distribution of three pelvic asymmetry measures for visual lameness grades 0 to 5 in *n* = 301 horses assessed by one board-certified specialist at a veterinary hospital in Switzerland. For each grade the asymmetry measures are summarised in the box plot as median (interquartile range) in mm: (**a**) AbPDMin, (**b**) AbPDMax and (**c**) AbPDUp.

Distribution of values of the three pelvic movement asymmetry measures for horses of lameness grades 0 to 5 are displayed in Figure 2. Grade 5 horses were excluded from further analysis due to an insufficient number of observations.

There was an overall difference in movement asymmetry between lameness grades for all three pelvic asymmetry measures (Kruskal–Wallis test, all *p* < 0.001). Post-hoc pairwise comparisons indicate that there were significant differences between the majority of paired lameness grades (Table 1). However, this was not always the case for adjacent lameness grades. For AbPDMin there was no significant difference for any adjacent grades, while for AbPDMax only adjacent grades 2–3 were significantly different and for AbPDUp only adjacent grades 0–1 were significantly different.

**Figure 3:**
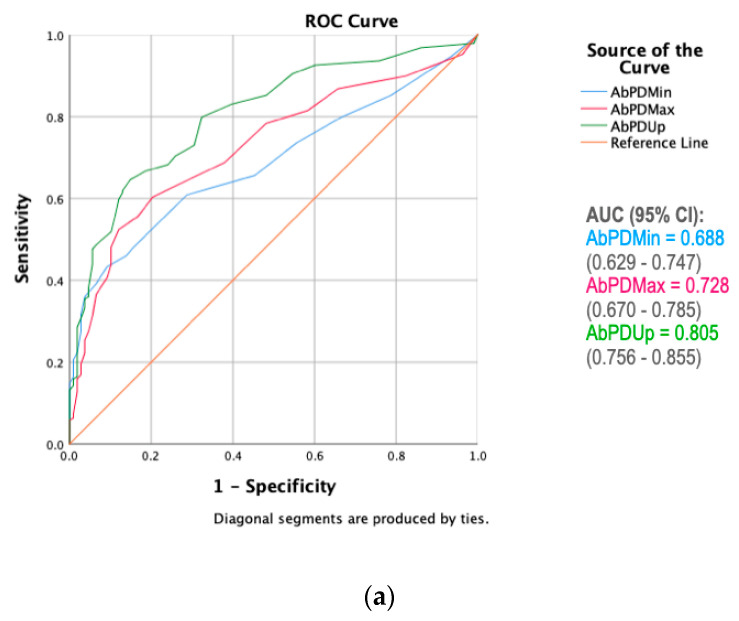

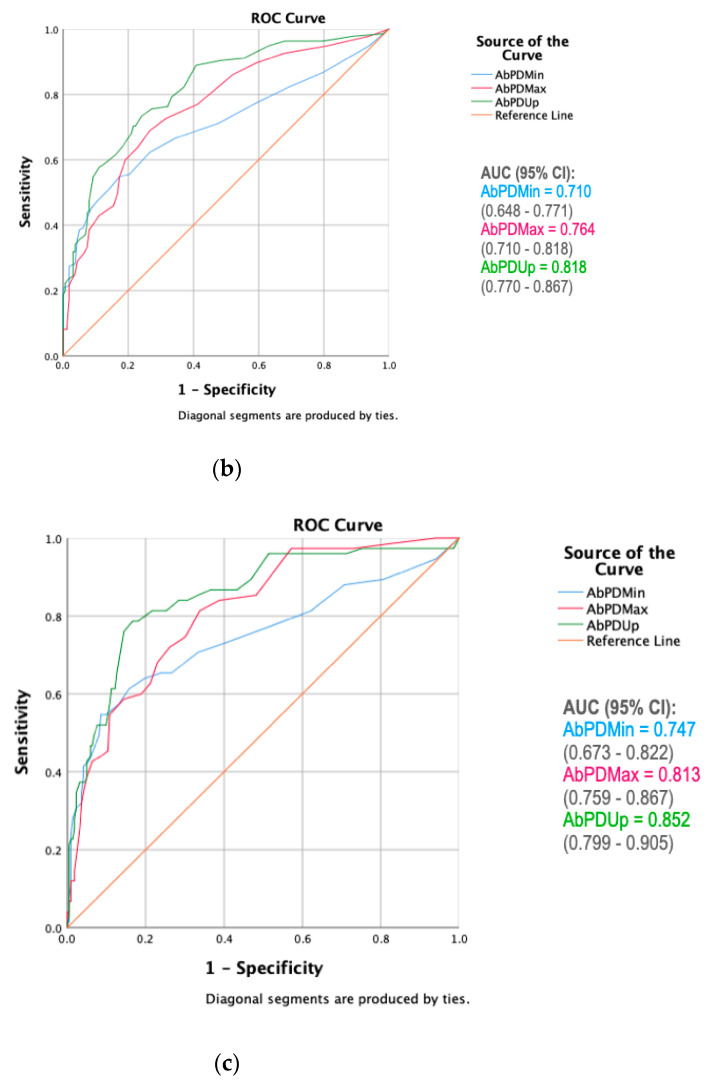

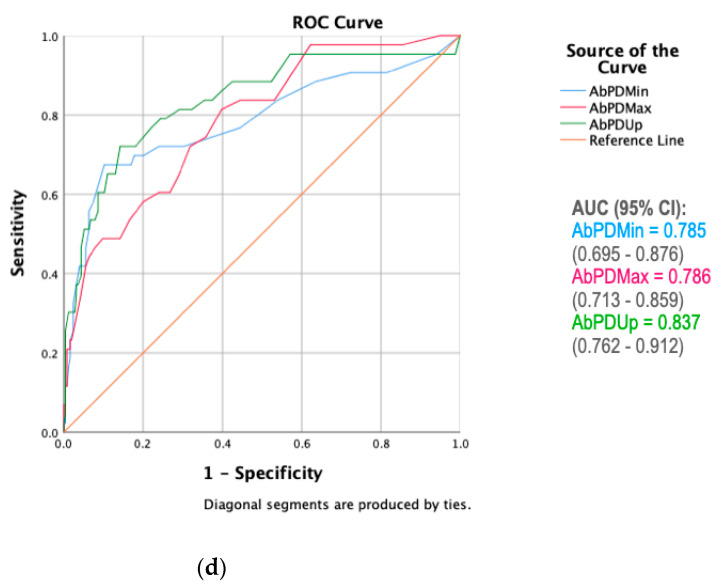
Receiver operating characteristic (ROC) curves for three pelvic asymmetry measures with calculated area under the curve (AUC) under 4 conditions: (**a**) discriminating between grade 0 (non-lame horses) and grades 1–4 (all lame horses), (**b**) discriminating between grades 0–1 and grades 2–4, (**c**) discriminating between grades 0–2 and grades 3–4 and (**d**) discriminating between grades 0–3 and grade 4.

Evaluation of the ROC curves (Figure 3) showed AbPDUp to be the measure with the highest discriminative power in differentiating between different lameness grades, i.e., it generally showed the highest sensitivity for any given level of specificity, across all asymmetry measures, with AUC values of 0.688–0.785 for AbPDMin, 0.728–0.813 for AbPDMax and 0.805–0.852 for AbPDUp.

Potential thresholds for differentiation between lameness grades were evaluated based on sensitivity and specificity for AbPDMin, AbDMax and AbPDUp and are shown in Table 2 (with minimum specificity benchmarks set at 75, 80 and 85%) and Table 3 (with minimum sensitivity benchmarks set at 75, 80 and 85%).

## 4. Discussion

This study investigated the clinical application of three pelvic movement symmetry parameters which can, for example, be calculated from a smartphone attached over the sacrum of a horse. Firstly, we investigated whether the average asymmetry values for the three pelvic movement symmetry parameters were significantly different between different lameness grades. Secondly, cut-off points were identified based on set minimum specificity or sensitivity requirements.

### 4.1. Comparison of Lameness Grades

Our results suggest that asymmetry values for all pelvic parameters, recorded by a smartphone, increase in line with higher lameness grades, as previously demonstrated by other studies both in forelimbs and hindlimbs [17,23]. It is interesting to note that the median symmetry values for each lameness grade increased in larger increments as the lameness grade increased. At 5 mm the median value for both AbPDMin and AbPDMax in horses classified with a grade 1 lameness in this study was marginally higher than the threshold of 3 mm [4,12], which was established by comparing objective symmetry measurements with the subjective lameness grade of a group of veterinarians. However, direct comparisons should be made with caution due to the use of a different system of grading lameness [24], in our case AAEP vs. UK scale, and due to the fact that different inertial sensor systems result in small differences in movement symmetry values [5,16].

Pair-wise comparisons revealed that in the majority of cases there were significant differences in the recorded objective parameters between different lameness grades. These differences were, however, often not significant for pairs of lameness grades which were just one grade apart—this was the case for grade 0–1 for AbPDMin and AbPDmax, grade 1–2 and 3–4 for all three pelvic asymmetry measures and grade 2–3 for AbPDMin and AbPDUp. Surprisingly, AbPDMin was a parameter which did not show significant differences between any two adjacent grades. Interestingly, AbPDUp was the only asymmetry measure with a significant difference identified between grade 0 (non-lame horses) and grade 1 (very mildly lame horses), with median values of 5 and 8.5 mm, respectively. Given the low agreement between experienced evaluators in horses with mild lameness [25], this parameter could prove valuable in a clinical setting where distinguishing between non-lame and mildly lame horses subjectively is often difficult. It might also be useful in the context of quantifying changes in symmetry following a treatment or to monitor progress during a rehabilitation program. However, it should be noted that our results might reflect the approach of this particular veterinarian, as it has been shown that different veterinarians utilise different strategies during visual lameness assessment—some focusing on upward movement [23], while others might focus on the downward movement of the pelvis.

### 4.2. Thresholds When Applying Minimum Specificity

When investigating three different pre-set minimum specificity benchmarks (75, 80 and 85%), the cut-off points for all three asymmetry measures increased with increasing lameness grade (by 1–4 mm from grade to grade for PDMin and PDMax and by 2–6 mm for PDUp, see Table 2). With a minimum specificity of 75%, the cut-off points for grade 1 (non-lame vs. lame) for both AbPDMin (6.5 mm at 54.4% sensitivity) and AbPDMax (7.5 mm at 60.3% sensitivity) exceeded the reported 3 mm asymmetry threshold [4,12]. However, they were similar to the values of 5.4 and 6.2 mm calculated from the correction equations for the thresholds for PDMin and PDMax, respectively, which accounts for differences between the systems used [16]. It is also worth noting that in a large population of riding horses (*n* = 222) with no reported lameness by the owner [14], the median values for PDMin and PDMax were also higher (7.9 and 9.6 mm, respectively, converted using equations from [16] to take into account the different systems used). With 85% specificity, the cut-off points identified in this study as 8.5 mm for PDMin (at 46% sensitivity) and 9.5 mm for PDMax (at 52.4% sensitivity) were similar to those previously reported in Thoroughbred racehorses (at 90% specificity: PDMin = 7.5 mm (at 90% sensitivity) and PDMax = 10 mm (at 50% sensitivity)) [18]. Our cut-off point for PDMin did, however, have a much lower sensitivity. Due to a lack of published data reporting PDUp values in clinically lame horses, there were no established asymmetry thresholds with which to compare the PDUp cut-off points reported here for the different lameness grades. However, for our set minimum specificity requirement the identified cut-off point between non-lame and lame horses was 10.5–12.5 mm (at 64.6–68.3% sensitivity). The higher cut-off points established in the present study for clinically lame horses and previously for Thoroughbreds in training [18], as well as the finding that most ‘owner-sound’ horses show asymmetries outside of the 3 mm threshold [14], indicate that further studies are needed to identify the influence of variation between groups of horses and/or groups of veterinarians in movement asymmetry in lameness [26]. In addition, the choice of technology (specific sensor and/or processing) may influence the agreement between measured parameters, highlighting the need to conduct validation studies for novel technologies [5,16].

### 4.3. Thresholds When Applying Minimum Sensitivity

Since the primary goal of the clinical lameness evaluation is to identify the (most) affected limb(s), it appears appropriate to focus on cut-off points with high sensitivity at the cost of lower specificity. For this reason, we also evaluated cut-off points for three minimum sensitivity benchmarks (75, 80 and 85%). For each given minimum sensitivity benchmark, the cut-off points increased by approximately 1–2 mm from grade to grade for AbPDMin and AbPDMax. For AbPDUp, they also increased between grades; however, less consistently (see Table 3). Even at the lowest sensitivity benchmark (75%), the grade 1 cut-off points, discriminating between non-lame and lame horses, for AbPDMin (2.5 mm, 33.3% specificity) and AbPDMax (4.5 mm, 51.9% specificity) were very close to the previously reported threshold of 3 mm [4,12]. This would suggest that when using a higher sensitivity (and hence lower specificity) in a lameness screening scenario, the gait analysis system, compared to the visual assessment of the clinician in this study, would tend towards classifying horses as lame. This was particularly the case for AbPDmin, where the 2.5 mm threshold (33.3% specificity, i.e., the proportion of non-lame horses correctly classified) would result in 66.7% of non-lame horses classified as lame, possibly resulting in welfare and economic implications. On the other hand, in the clinical setting, a high sensitivity is desirable, in particular when attempting to appreciate changes in asymmetry following diagnostic analgesia. However, when comparing these thresholds, it is important to note that different lameness grading systems were used [24], as well as different sensor systems, which could explain some of the differences in the movement symmetry values. For AbPDUp, the identified cut-off points between non-lame and lame horses were 5.5–7.5 mm (at 51.9–67.6% specificity). These thresholds are very close to the mean variation of this parameter (6 mm) recorded at different time points in horses judged as sound by an experienced veterinarian [22]. Therefore, asymmetry below this value could simply be due to natural variation.

### 4.4. Summary of the Discriminative Power of Pelvic Asymmetry Measures

It is important that an asymmetry parameter and the associated asymmetry thresholds can not only correctly identify lame horses (high sensitivity) but also that they do not incorrectly label non-lame horses as lame (high specificity). Out of the three investigated asymmetry measures, AbPDUp, an asymmetry parameter comparing the upward movement from the minimum position at mid stance to the maximum position in the aerial phase between the two halves of a trot stride, had the highest discriminative power (AUC) for all conditions under investigation. [5,16]. The results of the present study suggest that PDUp may be the most suitable measure for classifying hindlimb-lame horses as it maintained a high sensitivity (over 60%) even when the minimum specificity benchmark was set at 80%. A normalised version of this parameter (Symmetry Index Up) has also been shown to change in response to positive diagnostic analgesia in hindlimb-lame horses [20]. Our results suggest that AbPDMin is the parameter least able to discriminate between different lameness grades. This is surprising, since this parameter has been associated with differences in the peak force between contralateral hindlimbs [13], a kinetic parameter that has proven sensitive to hindlimb lameness [27]. PDMin has also been reported to show consistent change in response to successful diagnostic analgesia in horses with hindlimb lameness [20]. Finally, in a study in Thoroughbred racehorses comparing PDMin and PDMax to subjective lameness grading by a group of veterinarians [18], PDMin had a higher AUC (0.890) compared to PDMax (0.743). Whilst the latter finding might be a function of the specific group of veterinarians or the exclusive evaluation of Thoroughbreds in that study, the fact that AbPDUp is most reliable in our study may equally be a function of the approach to visual assessment of lameness used by this veterinarian. It has been suggested that some people may focus on upward movement [23], while others might focus on the downward movement of the pelvis. In addition, clinical studies in horses with hindlimb lameness [28,29] reported lower mean PDMin than mean PDMax values, suggesting that the changes in PDMin may be harder to identify visually or that the upward movement of the pelvis is more affected by lameness (resulting in a greater PDMax). Furthermore, only changes in PDMax were found to be significant after positive response to flexion tests when compared with a subjective judgement of a single veterinarian [30]. Repeating the study with more observers would be helpful to investigate how different factors, such as level of experience or training given, could help explain the differences.

### 4.5. Limitations of Smartphone-Based Pelvic Symmetry Measures

First, it appears important that users are trained appropriately in the correct placement of the smartphone [5] to avoid a directional bias in the measurements. Second, users should also be aware of the previously reported limits of agreement [5,16] when presented with gait analysis results recorded with different technological solutions. However, smartphone-derived symmetry measures might be useful for monitoring the success of treatment or rehabilitation programs, creating a quantitative record over time; however, this needs further investigations. Third, users of a single-sensor system need to be particularly aware of the compensatory effects of forelimb lameness on pelvic movement symmetry [31,32], since relative head withers movement cannot be consulted when deciding about the likely origin of a pelvic movement symmetry [33]. However, the compensatory movement changes in the pelvis in horses with induced forelimb lameness was reported to be of a smaller magnitude than compensatory changes in head movement resulting from induced hindlimb lameness [31]. Therefore, compensatory changes might be a bigger challenge in horses with hindlimb lameness.

## 5. Conclusions

In general, cut-off points calculated for quantitative measures of pelvic movement symmetry between lameness grades increased with the severity of lameness as scored subjectively by one veterinarian, and this is in line with previous studies. Contrary to our hypothesis, differences in the minimum position of the pelvis between the two halves of the stride cycle (PDMin) had the lowest sensitivity and specificity values, while the differences in pelvic upward movement amplitudes (PDUp) produced the highest values for sensitivity and specificity when using the subjective assessment of a single expert clinician. Although the use of a smartphone measuring only the symmetry of pelvic movement cannot replace a full lameness examination, it may present a useful adjunct, providing objective, quantitative values in addition to the subjective evaluation. Ultimately, it may also be a cost-effective method for documenting progression of lameness or improvements after treatment or during rehabilitation. This aspect deserves further attention.

## Figures and Tables

**Table 1:** *p*-values of post-hoc pairwise comparisons of lameness grades for three pelvic asymmetry measures. Values in **bold** highlight statistically significant results (*p* < 0.05, adjusted after Bonferroni correction).

Lameness Grades	AbPDMin	AbPDMax	AbPDUp
0–1	1	1	**0.02**
0–2	**0.02**	**0.001**	**<0.001**
0–3	**0.001**	**<0.001**	**<0.001**
0–4	**<0.001**	**<0.001**	**<0.001**
1–2	1	0.6	0.5
1–3	**0.2**	**<0.001**	**<0.001**
1–4	**<0.001**	**<0.001**	**<0.001**
2–3	1	**0.03**	0.08
2–4	**0.002**	**0.002**	**0.001**
3–4	0.4	1	1

**Table 2:** Threshold values for identifying horses in the specific lameness grade (or higher) under set minimum specificity benchmarks of 75, 80 and 85%.

Asymmetry Measure	Lameness Grade (AUC *)	Specificity Benchmark	Threshold ≥ (mm)	Sensitivity	Specificity
AbPDMin	Grade 1	75%	6.5	54.5%	77.8%
	(0.688)	80%	7.5	48.1%	84.3%
		85%	8.5	46.0%	86.1%
	Grade 2	75%	7.5	55.6%	79.6%
	(0.710)	80%	8.5	54.8%	82.7%
		85%	9.5	51.1%	85.8%
	Grade 3	75%	8.5	65.3%	76.1%
	(0.747)	80%	9.5	64.0%	80.2%
		85%	11.5	58.7%	86.0%
	Grade 4	75%	9.5	72.1%	76.0%
	(0.785)	80%	11.5	69.8%	82.3%
		85%	13.5	67.4%	86.6%
AbPDMax	Grade 1	75%	7.5	60.3%	79.6%
	(0.728)	80%	8.5	55.6%	83.3%
		85%	9.5	52.4%	88.0%
	Grade 2	75%	8.5	63.7%	77.2%
	(0.764)	80%	9.5	60.0%	80.9%
		85%	13.5	43.0%	88.9%
	Grade 3	75%	10.5	68.0%	77.0%
	(0.813)	80%	12.5	60.0%	81.1%
		85%	13.5	58.7%	85.6%
	Grade 4	75%	12.5	60.5%	76.0%
	(0.786)	80%	14.5	53.5%	83.5%
		85%	15.5	48.8%	85.8%
AbPDUp	Grade 1	75%	10.5	68.3%	75.9%
	(0.805)	80%	11.5	66.7%	81.5%
		85%	12.5	64.6%	85.2%
	Grade 2	75%	12.5	73.3%	75.9%
	(0.818)	80%	16.5	64.4%	81.5%
		85%	18.5	58.5%	87.7%
	Grade 3	75%	17.5	81.3%	78.4%
	(0.853)	80%	18.5	78.7%	82.0%
		85%	20.5	76.0%	85.6%
	Grade 4	75%	19.5	79.1%	75.6%
	(0.837)	80%	21.5	72.1%	81.9%
		85%	24.5	72.1%	85.8%

Sensitivity and specificity for three pelvic movement asymmetry measures in comparison to visual lameness scoring by one board-certified equine veterinary specialist. Grey colour identifies parameters with high sensitivity (i.e., 70% or more) or high AUC  * (above 0.8). * AUC: area under the curve.

**Table 3:** Minimum threshold values for identifying horses in the specific lameness grade (or higher) under set minimum sensitivity benchmarks of 75, 80 and 85%.

Asymmetry Measure	Lameness Grade (AUC *)	Sensitivity Benchmark	Threshold ≥ (mm)	Sensitivity	Specificity
AbPDMin	Grade 1	75%	2.5	79.9%	33.3%
	(0.688)	80%	1.5	85.2%	21.3%
		85%	1.5	85.2%	21.3%
	Grade 2	75%	3.5	77.0%	41.4%
	(0.710)	80%	2.5	82.2%	30.9%
		85%	1.5	86.7%	20.4%
	Grade 3	75%	4.5	77.3%	48.2%
	(0.747)	80%	3.5	81.3%	37.8%
		85%	2.5	88.0%	29.3%
	Grade 4	75%	5.5	76.7%	55.5%
	(0.785)	80%	4.5	83.7%	46.1%
		85%	3.5	88.4%	36.6%
AbPDMax	Grade 1	75%	4.5	78.3%	51.9%
	(0.728)	80%	3.5	81.5%	41.7%
		85%	2.5	86.8%	34.3%
	Grade 2	75%	5.5	77.0%	58.6%
	(0.764)	80%	4.5	85.9%	48.1%
		85%	4.5	85.9%	48.1%
	Grade 3	75%	7.5	81.3%	66.2%
	(0.813)	80%	7.5	81.3%	66.2%
		85%	5.5	85.3%	51.8%
	Grade 4	75%	7.5	81.4%	60.2%
	(0.786)	80%	7.5	81.4%	60.2%
		85%	4.5	97.7%	37.8%
AbPDUp	Grade 1	75%	7.5	79.9%	67.6%
	(0.805)	80%	6.5	83.1%	60.2%
		85%	5.5	85.2%	51.9%
	Grade 2	75%	11.5	75.6%	72.8%
	(0.818)	80%	8.5	82.2%	63.0%
		85%	7.5	88.9%	59.3%
	Grade 3	75%	20.5	76.0%	85.6%
	(0.852)	80%	17.5	81.3%	78.4%
		85%	12.5	85.3%	66.7%
	Grade 4	75%	20.5	76.7%	78.0%
	(0.837)	80%	19.5	81.4%	70.9%
		85%	12.5	86.0%	60.2%

Sensitivity and specificity for three pelvic movement asymmetry measures in comparison to visual lameness scoring by a board-certified equine veterinary specialist. Grey colour identifies parameters with high specificity (i.e., 70% or more) or high AUC * (above 0.8). * AUC: area under the curve.

## Data Availability

The data presented in this study are available in Supplementary Material S1.

## Supplementary Materials

The following are available online at https://www.mdpi.com/article/10.3390/ani11061665/s1, Table S1: Raw data: all movement values (mm).
